# The Rise of Gastrointestinal Cancers as a Global Phenomenon: Unhealthy Behavior or Progress?

**DOI:** 10.3390/ijerph20043640

**Published:** 2023-02-18

**Authors:** Silvia Rodrigues Jardim, Lucila Marieta Perrotta de Souza, Heitor Siffert Pereira de Souza

**Affiliations:** 1 Division of Worker’s Health, Universidade Federal do Rio de Janeiro, Rio de Janeiro 22290-140, RJ, Brazil; 2 Departamento de Clínica Médica, Hospital Universitário, Universidade Federal do Rio de Janeiro, Rua Prof. Rodolpho Paulo Rocco 255, Ilha do Fundão, Rio de Janeiro 21941-913, RJ, Brazil; 3 D’Or Institute for Research and Education (IDOR), Rua Diniz Cordeiro 30, Botafogo, Rio de Janeiro 22281-100, RJ, Brazil

**Keywords:** gastrointestinal cancers, lifestyle, syndemic, behavior, societal determinants

## Abstract

The overall burden of cancer is rapidly increasing worldwide, reflecting not only population growth and aging, but also the prevalence and spread of risk factors. Gastrointestinal (GI) cancers, including stomach, liver, esophageal, pancreatic, and colorectal cancers, represent more than a quarter of all cancers. While smoking and alcohol use are the risk factors most commonly associated with cancer development, a growing consensus also includes dietary habits as relevant risk factors for GI cancers. Current evidence suggests that socioeconomic development results in several lifestyle modifications, including shifts in dietary habits from local traditional diets to less-healthy Western diets. Moreover, recent data indicate that increased production and consumption of processed foods underlies the current pandemics of obesity and related metabolic disorders, which are directly or indirectly associated with the emergence of various chronic noncommunicable conditions and GI cancers. However, environmental changes are not restricted to dietary patterns, and unhealthy behavioral features should be analyzed with a holistic view of lifestyle. In this review, we discussed the epidemiological aspects, gut dysbiosis, and cellular and molecular characteristics of GI cancers and explored the impact of unhealthy behaviors, diet, and physical activity on developing GI cancers in the context of progressive societal changes.

## 1. Introduction

Gastrointestinal (GI) cancers, including stomach, liver, esophageal, pancreatic, and colorectal cancers (CRCs), represent more than a quarter of all cancers; moreover, their prevalence is continuously increasing [1,2]. In absolute numbers, pre-pandemic data estimated that in 2018, there were approximately 5 million new cases of GI cancers, with more than 3 million associated deaths [3]. Among the major malignancies of the GI tract, data obtained from the GLOBOCAN database for 2020 estimated that there were more than 600,000 new cases of esophageal cancer (EC), with more than 500,000 related deaths [4]. Using the same database, the global patterns of gastric cancer (GC) analysis estimated that there were 1.1 million new cases and more than 700,000 related deaths in 2020 [5]. Similarly, the incidence of colorectal cancer (CRC) has been rising at an alarming rate, with an estimated 1.9 million new cases and 900,000 related deaths worldwide in 2020 [6]. Although patients with CRC who are diagnosed at early stages usually have a relatively favorable prognosis [7], the increasing incidence of CRC among young adults over the last few decades [8,9] is a major concern. Even though the prognosis of GI cancers, in general, could be better among younger patients, the overall burden is greater, and the outcome can be worse than those of non-GI cancers [1]. The burden of GI cancers can be very significant in young patients given that they have a long life expectancy and constitute the major contributors to the economy and family support [10]. 

As recent studies indicate that most GI cancers share several common risk factors, such as smoking, alcohol ingestion, infections, dietary habits, and obesity [2,11], it has been hypothesized that their increasing incidence could be attributed to progressive changes in the presence of these factors [12,13]. Regarding CRC, it is important to highlight the decline or stabilization in the incidence of new cases in a small number of countries with a high human development index (HDI) [14,15], where CRC reduction has been attributed to relatively recent changes in lifestyle and the introduction of screening programs [2]. Therefore, in addition to advances in early detection and treatment options, there is an unmet and urgent need to invest in cancer research attempting to uncover basic mechanisms and the epidemiology behind the so-called modifiable risk factors.

Although breast, prostate, and lung cancers are highly prevalent, GI cancers rank first in terms of incidence and mortality and account for significant socioeconomic burden. Several environmental and lifestyle factors, such as smoking and alcohol consumption, have been associated with cancer development, including GI cancer [16]. Nevertheless, growing consensus also positions dietary habits as relevant risk factors for GI cancers [17,18,19]. Current evidence suggests that socioeconomic development results in several lifestyle modifications, including shifts in dietary habits to potentially less-healthy diets [14,20,21,22]. Moreover, recent data indicate that the increased production and consumption of processed foods underlie the current pandemics of obesity and related metabolic disorders, which are directly or indirectly associated with the emergence of various chronic noncommunicable conditions and GI cancers [23,24,25].

However, progressive environmental changes are not limited to those in dietary patterns. Unhealthy behavioral features are highly complex and should be analyzed with a holistic view of lifestyle. The incidence and mortality rates of GI cancers vary widely among different nations and even among different regions of each country. Apart from exposure to environmental factors, disparities in risk, prognosis, and survival of patients with GI cancers may depend much more on socioeconomic status (for example, access to prevention, vaccination, and early diagnosis) than on genetic or geographic risk factors [26]. Similarly, reducing health inequalities poses a challenge to most nations, where social determinants of health, from diet to access to screening, diagnosis, and proper treatment, differ considerably [27]. Understanding the disease risk and mortality of digestive cancers requires more than looking for genetic inheritance or individual habits, but very importantly, taking into account social, economic, cultural, and psychological circumstances that determine lifestyle and exposure to risk factors during all phases of life [28]. 

This review aimed to integrate available knowledge on the recent GI cancers generated in different fields through distinct methodologies, while analyzing the disease at the individual and the population levels in a holistic and complex framework. In this review, we discussed epidemiological aspects, genetic background, epigenetic modifications, gut dysbiosis, and cellular and molecular mechanisms shared by most GI cancers. Furthermore, we explored the impact of unhealthy behaviors, diet, nutrition, and physical activity on developing GI cancers in the context of the progressive globalization of socioeconomic and environmental structures. 

To address the question of the increase in the incidence of GI cancers as a global phenomenon and the potential role of unhealthy lifestyles, the methodology utilized in this study relied on a literature review using the MEDLINE database within the National Library of Medicine (PubMed). Herein, we propose a holistic approach for analyzing the several aspects potentially underlying the rise of GI cancers, considering the multidirectional interactions among the major individual components. Therefore, we combined a scoping approach to identify and analyze knowledge gaps, characteristics related to crucial concepts, and the types of available evidence in the most important GI cancer-related topics, with a narrative approach.

## 2. Epidemiology of the Major Gastrointestinal Cancers

### 2.1. EC

EC is the fourth most prevalent type of GI cancer. There are two primary histological subtypes of EC: adenocarcinoma and squamous cell carcinoma (SCC). Most patients with EC present with advanced disease; therefore, the mean overall 5-year survival rate is only 18% (10–30%). EC is thrice more frequent in male patients than that in female patients. The primary risk factors for SCC are alcohol and tobacco use, while those for adenocarcinoma are gastroesophageal reflux (especially erosive esophagitis and Barrett’s esophagus), tobacco use, and obesity. SCC is the dominant subtype worldwide, especially in Asia, Africa, and Southern Europe [29], whereas adenocarcinoma ranks first (nearly 60%) in the United States (US) and Northern Europe [30]. 

Nevertheless, considering the population of the US, adenocarcinoma accounts for nearly 68% of ECs in non-Hispanic whites, while SCC accounts for 80% of ECs in African Americans [31]. These differences could be explained by the interaction between multiple factors, as tenuous boundaries exist between ethnicity and lifestyle. Solving this complex equation requires paying attention to socioeconomic status and access to healthcare. For instance, functional variants in alcohol-metabolizing genes have been identified in Asian populations that, when associated with lifestyle factors, significantly increase the risk of SCC [32]. However, social and racial disparities in the incidence and mortality rates of GI cancers, including EC, as in other so-called noncommunicable and infectious diseases (such as the recent COVID-19 pandemic), have long been reported [33,34,35]. 

### 2.2. GC

GC is the fifth most common cancer and the third leading cause of cancer-related mortality worldwide. Noncardia GC (NCGC) accounts for almost 75% of GC cases [36]. Similar to other GI cancers, the epidemiology of NCGC varies greatly among populations, and outcomes are poor in most parts of the world; one of the reasons is probably its diagnosis at advanced stages [37]. Although the incidence of NCGC is significantly higher in East Asia (34 per 100,000 in South Korea; 28 per 100,000 in Japan) than that in Europe or the United States (6 per 100,000), Asian countries have reported that more than 60% of NCGC cases are diagnosed at a surgically or endoscopically curable stage. Although the incidence of GC has decreased in the US over the past decades, the rates of NCGC among people aged 50 years or less run in the opposite direction, and late diagnosis and poor outcomes are frequent. In addition to *Helicobacter pylori* infection, other major risk factors for GC include increasing age, male sex, nonwhite race, type of dietary intake, socioeconomic status, genetics, and smoking [38].

Chronic *H. pylori* infection leads to atrophic gastritis, followed by intestinal metaplasia of the stomach, and is considered a meaningful precursor lesion of GC [39]. Paralleling GC rates, *H. pylori* prevalence rates also vary widely among countries, from less than 40% in industrialized nations of Europe and North America to greater than 70% in South America, Africa, Eastern Europe, and East Asia [40,41]. In addition to geographical residence, ethnic disparities have been reported as relevant risk factors for GC. For example, in the US, although the overall incidence of GC is nearly 6 per 100,000 among the general population, some high-risk racial and ethnic groups (Asians, Alaskan Indians, American Indians, African Americans, Hispanics) may have a high GC risk [42].

### 2.3. CRC

CRC is the third most commonly diagnosed malignancy and the second leading cause of cancer-related death, with approximately 1.8 million new cases worldwide [6]. More than 90 percent of CRC cases occur sporadically, highlighting the importance of risk factors in addition to well-established cancer-related genes. Moreover, the global rise in CRC rates may be associated with environmental risk factors, such as unhealthy dietary patterns, overweight, obesity, type 2 diabetes, sedentarism, smoking, and alcohol consumption [43]. As observed in other GI cancer epidemiologic studies, CRC incidence varies widely between different countries and geographic regions, with Australia and New Zealand having the highest and South-Central Asia having the lowest rates [2]. 

Several studies have supported a relationship between the HDI and CRC incidence and mortality; the highest incidence rates are usually reported in developed countries. Even though economic development and the resulting industrialization are expected to improve access to healthcare, these greatly influence the adoption of the so-called Western lifestyle and the unhealthy habits mentioned above. Guided health policies and early access to healthcare services promote the improvement of CRC outcomes by implementing screening methods, detection, and removal of colonic polyps, as well as detection of early-stage CRC. In this complex and multifactorial context, recent studies have indicated that 49% of CRCs occur in very high HDI countries, with Europe and North America facing the highest CRC burden [44]. Nonetheless, incidence rates are increasing in numerous less-developed countries, and great differences in CRC rates among regions of large countries, such as Canada and Brazil, might be related not only to health policies, but also to regional deprivation and risk factors [45,46,47].

### 2.4. Liver Cancer (LC)

Primary LC can be broadly classified into hepatocellular carcinoma (HCC) and intrahepatic cholangiocarcinoma (ICC), which are responsible for 75–85% of cases. Poor prognosis is a hallmark of the disease; therefore, the incidence and mortality patterns of LC are closely aligned, as LC ranks third both in incidence and mortality among GI cancer cases. Further, LC is the sixth–seventh most commonly diagnosed cancer and the fourth leading cause of cancer-related deaths worldwide [48]. The global distribution of LC varies widely, with nearly 75% of cases occurring in Asia, with China accounting for over 50% of cases and Mongolia having the highest incidence (93.7 per 100,000). In the last four decades, trends of increasing LC cases have been noted in some countries, such as the US, Canada, New Zealand, and Australia [2].

Hepatitis B virus (HBV) and hepatitis C virus (HCV), along with alcohol consumption, are considered the most important risk factors for HCC worldwide. Cofactors for HCC in HBV and HCV carriers include male sex, long duration of infection, HCV genotype 3, coinfections with HIV, insulin resistance, and tobacco use. As obesity, diabetes, and the associated metabolic syndrome have become highly prevalent, nonalcoholic fatty liver disease is considered one of the most common causes of chronic liver diseases and a relevant cause of HCC [48]. Developing nations face a peculiar challenge, as the industrialization process influences the socioeconomic environment, and lifestyle changes are noticed, favoring a high-caloric Westernized diet and increasing the rates of obesity and diabetes [49]. The high male prevalence of HCC can be attributed to exposure to risk factors, such as viral hepatitis, alcohol abuse, cigarette smoking, and elevated iron storage [50]. 

### 2.5. Pancreatic Cancer (PC)

PC is the least common of the five major GI cancers and the twelfth most common of all cancers, with a cumulative 5-year survival rate of only 5–15% [51]. Moreover, predicted mortality trends of PC in the next decade are not favorable [52]. Both the incidence and mortality rates of PC are 3–4-fold higher in high HDI countries, with the highest reported rates in North America, Europe, and Australia/New Zealand, and rates in male patients being slightly higher than those in female patients [2]. Higher rates of PC in men could be related to higher exposure to environmental and social risks, such as tobacco and alcohol [53]. A few additional hypotheses could explain, at least to some extent, these findings: the adoption of a Westernized lifestyle, which includes the consumption of processed food, red meat, high sugar, and fat meals; global increase in metabolic risk factors, such as high body mass index (BMI) or increased bone mineral density, and alcohol consumption, which might also be related to the increase in the urbanization process; and equally significant, proper access to health care [54,55]. PC is usually highly aggressive and hard to diagnose due to nonspecific clinical manifestations. Moreover, diagnostic accuracy varies widely among countries and regions of the same country, depending on access to tertiary healthcare units, chiefly associated with urban, metropolitan, and highly developed centers [56]. BMI, type 2 diabetes, and, of utmost importance, alcohol and cigarette smoking are modifiable risk factors for PC. Regarding the risk of PC, an odds ratio of 1.74 (95% CI 1.61–1.87) was found for current smokers compared to nonsmokers. Heavy alcohol intake seems to be associated with PC and is a relevant cause of pancreatitis, an established risk factor for PC [57]. 

## 3. Lifestyle and Risk Factors for Gastrointestinal Cancers

### 3.1. Alcohol

Alcohol use is a leading risk factor for the global disease burden, and alcohol consumption can increase the risk of cancer. Between 1990 and 2017, global adult per capita alcohol consumption increased, the prevalence of current drinking increased from 45% to 47%, and lifetime abstinence decreased from 46 to 43%, and both trends are forecasted to continue by 2030 [58]. Educational status, diet, tobacco use, personal preferences, and regional and religious habits are some of the multiple lifestyle factors associated with patterns of alcohol use or abstinence and may confound the current research results. Alcoholic beverages cause nearly 4% of cancers, and the highest risk is associated with heavy alcohol consumption. Considering the different patterns of drinking, studies have shown varied associations between cancer risk and drinking frequency, quantity per usual drinking day, and heavy episodic drinking, all of which are associated with increased risk [59].

Several GI cancers have been associated with alcohol consumption. Squamous cell EC, but not esophageal adenocarcinoma, is associated with alcohol consumption. Studies have shown varying results regarding CRC and PC; the risk for PC seems to be related to heavy drinking, and the risk for CRC is associated with moderate or heavy drinking. Regarding GC, the World Cancer Research Fund/American Institute for Cancer Research—2018 report observed an increased risk in patients with an alcohol intake of >45 g/day [60]. HCC is directly linked to alcohol consumption; the association is mainly driven by alcohol-related cirrhosis, alcohol consumption in carriers of hepatitis B or C virus, and probably heavy drinking [60,61]. Alcohol might foster carcinogenesis through several pathways, including the following: both ethanol and its metabolite, acetaldehyde, can impact DNA methylation, leading to the expression of oncogenes; acetaldehyde forms DNA adducts that impair DNA synthesis and repair and cause mutations; inflammation, induction of oxidative stress, disruption of folate absorption, reduced function of the immune system, dysbiosis of the microbiome, liver cirrhosis, and changes in estrogen regulation may also play a role in cancer development [62].

### 3.2. Tobacco

Smoking is a major risk factor for several diseases, including GI cancers. Although tobacco smoking rates have declined in recent decades, smoking-associated diseases and deaths remain a matter of great concern and a global health problem. The Global Burden of Diseases, Injuries, and Risk Factors Study 2019 identified tobacco as the leading factor among 87 risk factors in terms of disability-adjusted life-years in men and the seventh in women [53]. Tobacco smoke contains different chemical agents, including reactive oxygen species (ROS) and reactive nitrogen species (RNS). Oxidative damage leads to genetic and epigenetic alterations, gene dysregulation, disruption of regulatory elements, and activation of inflammatory response pathways that, in a vicious cycle, result in further generation of ROS and may ultimately evolve into cancer initiation and progression [63].

Several studies over the last few decades have supported the relationship between smoking and GI cancer. Tobacco smoking has also been associated with a 20–30% increase in the risk of esophageal SCC, and importantly, a positive synergistic effect of combined tobacco and alcohol use has been noted [64]. The data also suggest that smoking is a risk factor for both cardia and noncardia GC. As reported for other GI cancers, smokers with a higher rate of cigarette consumption are at a higher risk of GC [65,66,67]. Meta-analyses also support the role of smoking in CRC development [26,68]. Cigarette smoking is a well-established risk factor for LC and PC. The 2014 US Surgeon General’s report presented an increase in the risk of LC derived from tobacco smoking of 70% for current smokers and 40% for former smokers [69]. The risk of PC is the highest among those who smoke the greatest number of cigarettes daily. Meta-analytical studies have found an elevated odds ratio for PC in current smokers compared to that in nonsmokers, but higher for heavy smokers, which decreases proportionally with years after cessation [70,71,72]. 

Quitting smoking and controlling tobacco consumption demand strategic planning. Lifestyle adoption seems to transcend people’s choices. Successful smoking cessation attempts have been related to socioeconomic status, level of education, access to protobacco advertising, antitobacco campaigns, and living with other smokers. Knowledge of sociodemographic data is relevant in guiding the implementation of public health policies, as the benefits of smoking cessation are well established. Former smokers show a reduced risk of death and cancers [73,74].

### 3.3. Physical Inactivity, Overweight, and Obesity

A substantial component of public health targets modification of lifestyle and environmental risk factors. The Global Burden of Diseases, Injuries, and Risk Factors Study (GBD) 2019 concluded that from 2010 to 2019, consistent declines (annual rate of change larger than −0.5%) were noted with respect to risks strongly associated with socioeconomic development, measured by the Socio-Demographic Index (SDI), such as household air pollution, unsafe water, sanitation, and handwashing. Tobacco smoking, a risk factor showing a trend of substantial decline, is not necessarily associated with a low SDI. On the contrary, tobacco smoking might increase as countries increase their SDI, at least temporarily. Many exposures that increase by more than 0.5% per year are metabolic risk factors, including high BMI and alcohol use [53]. 

Although economic development parallels better healthcare, metabolic risk factors could be related to an increase in the urbanization process, which promotes sedentary jobs and inactive lifestyles. Many individuals spend more than half of their time awake while performing sedentary tasks [54]. A systematic review emphasized that, despite variations by region, low and high socioeconomic groups in most low-income and low–middle-income countries present different health risks associated with lifestyle. Tobacco, alcohol, and red meat consumption was more prevalent in low socioeconomic groups, which also displayed less consumption of fruits, vegetables, fish, and fiber, than high socioeconomic groups. High socioeconomic groups tended to have higher levels of physical inactivity and consumed more fats, salt, and processed foods than low socioeconomic groups [75]. An increased BMI should be considered as an adjunct to physical inactivity, excess caloric intake, and diet quality. Obesity is an established risk factor for 13 different cancers, including major GI cancers. Physical inactivity, sedentary behavior, and obesity are probably related to cancer incidence via biological pathways, including insulin sensitivity, sex steroids, metabolic hormones, and chronic inflammation [53]. 

### 3.4. Infections

Malignancy develops in a multistep process, and bacteria and viruses have been identified as tumor promoters. Tumor promoters stimulate signaling pathways and cellular proliferation, which can ultimately lead to cancer. For example, infection with *H. pylori* accounts for more than 60% of GC, and its prevalence varies widely, from 25–50% in developed countries to 70–90% in developing countries [76]. The recurrence of *H. pylori* infection is very high in developing countries, either due to recrudescence or reinfection. In addition to the prevalence of *H. pylori* infection, living conditions, economic development, and health conditions have also been associated with infection recurrence [38,77].

Infections from HBV and HCV are currently the most important global risk factors for HCC, which is the main histological type of LC. Accordingly, patients from areas with high HCC prevalence rates tend to be younger than those with low HCC prevalence rates at diagnosis [78]. HBV is a DNA virus, and the evaluation of tissue tumors has commonly shown HBV DNA integration into the genome [48]. The resulting chronic necroinflammatory disease from HBV induces mutations in liver cells, and the lifetime risk of developing HCC is estimated to be 10–25% and is dependent on the presence of active HBV infection and/or cirrhosis. HCV is an RNA virus that does not integrate into its host genome. Tumorigenesis by HCV is probably the result of repetitive damage, regeneration, and fibrosis, and nearly 90% of HCV-associated HCCs are preceded by cirrhosis [48].

## 4. Common Cellular and Molecular Mechanisms

### 4.1. Genetic Susceptibility and Epigenetic Modifications

Although cancers present marked variability in tissue origin, histopathological subtypes, and clinical outcomes, they all result from acquiring heritable genomic modifications in the mutant cells comprising the tumors [79]. Abnormalities in critical homeostatic events, such as chromosomal instability, alterations in the methylation frequency of CpG islands in the promoter regions of cancer suppressor genes, and instability of microsatellite DNA regions disrupt the cancer-associated genes. Consequently, such changes induce alterations in the cell cycle, fundamentally disturbing several cellular functions, including proliferation, invasion, migration, and signaling [80]. Nevertheless, the process underlying temporospatial clonal evolution is also crucial for the development of cancer, which is thought to evolve following a stepwise accumulation of a series of genetic and epigenetic abnormalities in normal tissue [81]. In this context, during neoplastic development and malignant transformation, the tissue microenvironment is believed to produce a variable selective pressure that determines the favorable phenotypic attributes that ultimately allow the establishment of the tumor. 

Several susceptibility genes have been shown to influence the development of malignant neoplasms of the digestive system. For example, genes categorized as having high-penetrance, such as *MHL1*, *MSH2*, *MSH6*, *PMS2*, and *EPCAM* (related to Lynch syndrome), have been primarily associated with CRC, but they are also with other cancers, such as gastric and pancreaticobiliary cancers [82]. The *APC* gene (related to adenomatous polyposis syndromes) predisposes patients to CRC in up to 100% of cases and is also associated with gastric and small-bowel cancers [83]. *TP53* (Li-Fraumeni syndrome) [84], *BMPR1A,* and *SMAD4* (juvenile polyposis syndrome) predispose to CRC and GC. *STK1* (Peutz–Jeghers syndrome) predisposes patients to several tumors, including CRC, PC, GC, and small intestinal cancer [83]. However, only a relatively small percentage of tumors can be attributed to well-established cancer-related genes [85,86,87,88,89], which does not explain the worldwide increase in the incidence of GI cancers. 

Epigenetics, characterized by heritable modifications in gene expression not followed by permanent changes in the DNA sequence, plays a central role in the pathogenesis of various cancers [90]. Epigenetic changes represent an important example of how the effects of exposure on genes can influence disease development. Disruption of any of the intrinsic processes involved in normal epigenetic regulation may result in abnormal activation or silencing of genes that are frequently associated with cancer [91]. For example, hypermethylation of the promoter of the DNA repair gene *MHL1* determines microsatellite instability, which has been typically linked not only to CRC [92], but also to several GI cancers, including GC, PC, esophageal adenocarcinoma, and HCC [93]. The Wnt signaling pathway is a crucial cascade for tissue homeostasis and regeneration, and its dysregulation, mediated by hypermethylation [94] or microRNAs [95], has been associated with cancer development and affects the tumor microenvironment and immune response. Abnormal activation of the Wnt signaling pathway through genetic and epigenetic modifications has been associated with cancer progression and poor prognosis in several tumors, including CRC, GC, PC, EC, and LC [96]. 

### 4.2. Carcinogenic Pathways

Several signaling pathways and molecular networks have been implicated in carcinogenesis. The maintenance of tumorigenic properties of cancer cells depends on functional modifications of specific genes and signal transduction mediated by the binding of ligands to specific cell receptors. The preferential use of aerobic glycolysis, which produces lesser ATP than aerobic respiration (known as the Warburg effect), highlights metabolic reprogramming as a hallmark of cancer. Dysregulation of cellular energy metabolism constitutes an early event in carcinogenesis and positions mitochondrial function as fundamental for cancer cells [97]. The shift from oxidative to predominantly glycolytic metabolism often requires the activation of the phosphatidylinositol 3-kinase (PI3K)/AKT/mammalian target of the rapamycin (mTOR) pathway, one of the most ubiquitous abnormalities in cancer [98]. PI3K/AKT/mTOR activation upregulates glycolytic and lipogenic genes and stimulates enzymes to drive glycolysis, converting most of the resulting pyruvate into lactate [99]. Activation of MYC induces glutaminolysis, supplying substrates to the mitochondrial tricarboxylic acid cycle, resulting in citrate production, and ultimately acetyl-CoA for lipid biosynthesis and protein modifications [97]. Additionally, abnormal mitochondrial metabolism can determine the overproduction of ROS, with consequences on transcription factors, including HIF1-alpha and FOS-JUN, resulting in cancer cell proliferation [100,101]. 

The major cascades recognized for their critical roles in cancer are linked to each other and other intracellular signal transduction pathways, mediating upstream signals from receptor tyrosine kinases (RTK) [102]. For example, the epidermal growth factor receptor, a transmembrane RTK, plays an essential role in epithelial cell proliferation, differentiation, and survival, and its overexpression is associated with poor prognosis in CRC [103] and GC [104]. KRAS, a member of the Ras family of small GTPases, is involved in the activation of signal transduction pathways, such as the RAS/RAF/mitogen-activated protein kinase (MAPK) pathway, c-RAF/MEK/ERK, and PI3K/AKT, which regulate cell proliferation, survival, and differentiation [105]. KRAS is regarded as a major oncogenic promoter in various cancers, and KRAS mutations have been frequently detected in PC and CRC [106]. 

Chronic inflammatory diseases, including inflammatory bowel disease, Barrett’s esophagus, chronic pancreatitis, chronic gastritis, chronic hepatitis, and nonalcoholic steatohepatitis, have all been associated with an increased risk of cancer development. One of the major links between inflammation and cancer has been attributed to NF-κ B, a transcription factor associated with cell proliferation, apoptosis, and angiogenesis, which is activated by various stimuli, including inflammatory mediators, growth factors, and microorganisms [107]. NF-κB regulates the production of several inflammatory mediators, including the IL-6 family of cytokines [108], involved in the promotion of tumor development through Stat3 signaling, particularly in the early stages of colitis-associated CRC [109]. Another key protumorigenic mechanism involved in the NF-κ B pathway is the activation of antiapoptotic gene expression, which inhibits apoptosis induced by proinflammatory cytokines, such as TNF-α [110]. 

Other redundant and overlapping pathways also contribute to the inflammatory environment, which favors cancer development. For instance, the Janus kinase (JAK)/signal transducer and activator of transcription (STAT) pathway mediates proinflammatory gene expression and transcription and can also signal PI3K protein (PI3K/Akt pathway) and Ras protein (RAS-MAPK pathway) [111]. Nevertheless, even in disorders associated with low-grade inflammation, such as obesity, the inflammatory milieu maintained by proinflammatory cytokines constitutes an important mechanism underlying carcinogenesis. In a state of metabolic imbalance, particularly in obesity, inflammatory cytokines, immune mediators, tissue damage, adipocytokines derived from adipose tissue, and the resultant activation of NF-κ B and other signaling pathways combine to generate a carcinogenic environment [112]. 

The epidemiological association between obesity and cancer, including GI cancers, has been proposed to stem from several intricate mechanisms, such as insulin resistance, hyperinsulinemia, oxidative stress, chronic inflammation, and adipocytokine production [113]. Leptin, a hormone predominantly produced by adipose cells, has been associated with obesity [114] and has also been implicated in the development and prognosis of GI cancers, including CRC [115,116], probably because of its ability to promote the enhancement of cell proliferation and migration, inflammation, and antiapoptotic pathways [117]. Moreover, leptin synergizes with several oncogenes, cytokines, and growth factors converging into a downstream cascade, including the JAK-2/STAT, MAPK/ERK, and PI3K/AKT pathways [118,119]. 

The worldwide association of increasing metabolic disorders, including obesity and diabetes, with noncommunicable diseases, such as cardiovascular diseases and cancer, has recently been attributed to lifestyle changes, including the contemporary abundance and availability of food [120]. It has been hypothesized that nongenetic transmission may underlie obesity and insulin resistance [121]. It has been postulated that the effects of diet could be transmitted epigenetically to offspring, reinforcing the idea that chromatin represents a sensor and a mechanism by which metabolic changes are converted into stable patterns of reprogrammed gene expression [122]. Evidence supporting the link between diet and metabolism and disease is further corroborated by the characterization of specific metabolic sensors and their functions. For instance, mTOR modulates protein synthesis, insulin signaling, and mitochondrial function [123]. Adenosine monophosphate (AMP)-activated protein kinase (AMPK), a sensor of cellular energy status, regulates metabolic pathways that restore energy homeostasis [124]. Thus, it has been proposed that nutrient-based overabundance of metabolites and inflammatory products regulates gene expression via epigenetic modifications [125]. Nonetheless, in addition to typical markers of metabolic health, such as insulin regulation, waist circumference, and BMI, body metabolic rate (BMR), which reflects whole-body energy metabolism, has also been proposed as a relevant risk factor for cancer. Recently, in a large prospective European cohort, long-term follow-up identified a positive association between BMR and several cancers, including CRC, PC, and esophageal adenocarcinoma. The increased incidence observed, even among normal-weight individuals, appears to identify a subgroup of the population at great risk of these types of cancer, independent of adiposity [126]. 

Chronic psychological stress has also been implicated as a risk factor for the development of several diseases, including cancer [127,128,129]. Chronic stress stimulates the hypothalamic–pituitary–adrenal axis and sympathetic nervous system, leading to the synthesis of stress-related mediators and activation of the renin–angiotensin system [130]. Excessive production of corticosteroids and catecholamines induces the production of proinflammatory cytokines and metabolic changes, including an increase in insulin resistance and the release of free fatty acids from lipolysis [131]. Taken together, these alterations appear to create an inflammatory environment that favors the pathogenesis of metabolic syndrome, diabetes, and insulin resistance and the development of other noncommunicable chronic and immune-mediated diseases, all potentially mediated by chronic psychological stress [132,133]. In cancer, adrenergic receptors are overexpressed in neoplastic cells and the tumor microenvironment [134]. The downstream activation of adrenergic receptors, in turn, inhibits apoptosis and DNA repair with proto-oncogenic effects that enhance cell cycle progression [135]. Activation of adrenergic receptors induces the PI3K/AKT signaling pathway, with consequent stimulation of cell proliferation and angiogenesis [136]. In addition, stress-mediated alterations in the inflammatory response and immune function might compromise immune surveillance mechanisms, further favoring carcinogenesis [137]. 

### 4.3. Unhealthy Diet, Gut Dysbiosis, and Other Environmental Triggers

Currently, the gut microbiome is regarded as a physiological organ with a moldable composition that dynamically varies according to age and shifts according to various environmental exposures, including dietary patterns [138]. With the advent of technological advancements, including next-generation sequencing and metabolic profiling, a considerable amount of data has allowed a good understanding of how diet shapes the gut microbiome and may affect health and disease [139]. Interindividual variation in the gut microbiome has been attributed primarily to diet, but it is also attributed to environmental factors, such as lifestyle, exposure to pollutants, use of antibiotics, and, to some extent, host genetics [140,141]. Nevertheless, in a large population-based study, whole-microbiome composition was significantly more influenced by cohabitation than that by genetics. A healthy microbiome pattern was associated with a healthy diet, exposure to rural environments and pets, green spaces, and a high income. In contrast, reduced exposure to diverse microbiota favors an increase in the frequency of immune-mediated inflammatory and allergic diseases [142]. Further, a Western diet, rich in carbohydrates and fat, has been associated with a remarkable reduction in gut microbiome diversity [143]. 

The global increase in CRC, the most common GI tumor, has been largely associated with risk factors regarded as the Westernization of lifestyle. Such widespread lifestyle changes have been attributed to industrialization and economic development, generally accompanied by dietary modifications, including increased consumption of sugar, refined grains, fat, processed food, and fewer vegetables [8,43]. Therefore, in modern societies, where there is usually an accumulation of risk factors for CRC, such as high levels of sedentarism, smoking, alcohol consumption [144], type 2 diabetes [145], and obesity, an increase in the incidence of CRC is expected [146,147]. The positive association between CRC and regular ingestion of red and processed meat has been consistently reported for over a decade [24]. Heterocyclic amines and polycyclic aromatic hydrocarbons from high-temperature cooking [148,149] and nitrates and nitrites used in meat processing [150] could all favor the development of CRC. In particular, dietary heme iron intake from red and processed meat has been associated with CRC via the generation of potentially carcinogenic N-nitroso compounds [148]. 

Progressive advancements in food processing and agroindustrial production have led to the expansion of food products that contribute to Westernized menus [151]. These Westernized dietary products, which are usually ultra-processed and with high contents of sugar, saturated fat, and salt, but low in fiber and micronutrients [152], have been associated with cardiovascular diseases, metabolic syndrome, obesity, and overall cancer, among other health disorders [153]. Recent data from large prospective population-based cohorts identified a significant association between high consumption of ultra-processed foods (UPF) and an increased risk of CRC [154] and PC [155]. In addition, in a case-control study, the association between UPF intake and the risk of CRC increased but remained constant after adjustment for BMI, physical activity, educational level, type of job, and income [156]. In another large cohort study, the long-term follow-up of individuals consuming highly proinflammatory diets was associated with an increased risk of developing CRC, which was compensated by fiber ingestion [157]. Recent data from a large developing country showing a temporal association of increasing CRC with overweight, obesity, and diabetes indicate the progressive Westernization of the lifestyle in that country [45] in which dietary habits have been shifting from a traditional fiber-rich diet to a predominant high-calorie diet [158]. Whole grain intake has also been associated with a reduced risk of CRC [159], an effect that has been attributed to the reduction in bowel transit time and increased production of short-chain fatty acids, including butyrate, with anti-inflammatory [160] and anticancer properties [161]. 

An imbalance in microbial populations and certain gut microbiota components has been associated with the development of diseases, including GI cancers [162,163]. For example, infection with *H. pylori* has long been associated with the development of atrophic gastritis, metaplasia, and dysplasia, and their progression to cancer [164]. The carcinogenic potential of *H. pylori* is further corroborated by therapeutic eradication strategies that significantly reduce the risk of cancer development [165]. Recent data also support the role of gut microbial imbalance in the development of CRC. In a recent large meta-analysis, investigators found several consistent taxonomic differences in the gut microbiota with respect to CRC. For example, CRC tumor biopsies revealed a high abundance of the phylum *Fusobacteria*, whereas other studies found high levels of *Fusobacterium* and *Fusobacterium nucleatum* in mucosal samples of patients with CRC [166]. In addition, several studies have provided consistent data regarding the abundance of *Parvimonas*, *Porphyromonas,* and *Peptostreptococcus* in fecal and biopsy samples from patients with CRC [167]. The abundance of other intestinal bacteria, including *Bacteroides*, *Akkermansia*, and *Ruminococcus,* has also been implicated in the development or progression of CRC by inducing a proinflammatory milieu [168]. Similarly, the enrichment of *Proteobacteria*, including the genus *Campylobacter* [169] and the species *E. coli* [170], in the intestinal microbiome has also been associated with the development of CRC by modifying the tumor microenvironment. Moreover, in a large database case-control study, antibiotic use over previous years was associated with subsequent CRC development, reinforcing the potential carcinogenic effects of gut dysbiosis [171]. 

In PC, *Gammaproteobacteria* found in tumors have been suggested to be translocated from the gut [172]. The idea that gut bacteria might play a role in pancreatic tumor development was further corroborated by the demonstration of bacterial translocation in an experimental model [173]. Liver carcinogenesis is also associated with the gut microbiome. It has been postulated that gut microbial metabolites, such as secondary biliary acids and microbe-associated molecular patterns, enter the liver through the portal vein and contribute to carcinogenesis. Such effects are mediated by the activation of TLR4 with consequent overexpression of hepatomitogens and epiregulin [174]. Biliary acids, in turn, have also been implicated in liver carcinogenesis, as they induce prostaglandin E2 and cyclooxygenase-2 cascades, which promote tumor development [175] and inhibit NK cell recruitment, further contributing to cancer immune evasion [176].

## 5. Influence of the Exposome on Gastrointestinal Cancers

### 5.1. Multifactorial Origin and a Complex Network of Interactions

Functional interactions among diet, intestinal microbiota, and host homeostasis are complex and appear to involve epigenetic programming. In an experimental model, microbial constituents were shown to regulate histone acetylation and methylation in host tissues according to dietary patterns. For example, the consumption of a Western-type diet hampers many chromatin changes that occur in a plant-based diet. Nonetheless, supplementing germ-free mice with short-chain fatty acids could reverse chromatin modification states and transcriptional responses imposed by the gut microbiota on host epigenetic programming [177]. In addition to a long-term diet imbalance, epigenetic regulation has been associated with other factors, such as the gut microbiota and physical activity [178,179]. Modifications in gut microbial composition may affect epigenetic patterns, which orchestrate multiple molecular and cellular homeostatic processes in a potentially reversible fashion. The proposed mechanisms by which the microbiota induces immune and metabolic changes are, for example, signaling via Toll-like receptors, including NF-κ B activation or the signaling of microbiota-derived short-chain fatty acids via G-protein coupled receptors, and histone deacetylases that may be involved in the development of metabolic disorders [180] and CRC [181]. Among the commonly investigated associations between cancer and lifestyle factors, physical activity also plays an important role [182]. Although the exact mechanisms by which physical activity reduces the risk of CRC development are not completely understood, it has been recently suggested that exercise-induced changes in the gut microbiota might play an important role [183]. 

In the last century, multiple geosocial factors have been linked to the global rise in noncommunicable diseases, including cancer and chronic immune-mediated inflammatory diseases, in parallel with cardiovascular diseases and metabolic disorders. Among these factors, common societal transformations, usually involving socioeconomic development or industrialization, have been followed by progressive urbanization, resulting in densely populated cities. Several other factors have contributed to the complex context of socioeconomic changes, including increased industrial activities, changing working conditions, increased pollution, decreased biodiversity, changes in households and workplaces, family structure, and population composition, with increased migratory movements and the presence of refugees. 

Considering the concomitant presence of factors usually shared among most chronic noncommunicable diseases [184], such as the microbiome, genetic, and epigenetic modifications, it is conceivable to think of an increase in GI cancers as part of a dynamically changing exposome; however, they are progressively very globalized and less diverse. Communities still living according to traditional lifestyles harbor a similar microbiota composition compared with those residing in highly developed and industrialized societies [185,186]. Considering that the microbiota has coevolved with humans over millions of years, it is believed that commensal microorganisms greatly contributed to shaping our biology [187]. Nevertheless, the reduced biodiversity found in the gut microbiota of contemporary industrialized societies might reflect not only the progress in medicine, the use of antibiotics, sanitation, and dietary changes, but also the progressive reduction in environmental biodiversity [188]. As the Westernized or industrialized lifestyle spreads globally, changes in the microbiota, constituting an industrial or Westernized gut microbiota, are accompanied by a proinflammatory profile that underlies the rise of most noncommunicable chronic diseases, including cancers. Therefore, an improved understanding of the multidirectional interaction between macro-ecosystems with humans and their gut microbiota and the effects of microbiota dysfunctions induced by different lifestyle aspects may greatly help prevent diseases. 

### 5.2. Anthropocene, Social Connectivity, and Metacommunity 

The idea that Western lifestyle factors may trigger gut microbiota, genetic, and epigenetic modifications has recently been regarded as a potential explanation for the rise in GI cancers and their clinical presentation and outcomes, especially in the urban context. Hence, identifying such factors is a critical step in the prevention of GI cancers. Regarding this, it is important to consider the exposome not only as a measure of individual exposures in a lifetime, including insults from environmental and occupational sources to genetics, epigenetics, immune system, and microbiota, but also as an impact to our health. This should position humans as mere victims of a hostile and almost static environment. In fact, human activities have promoted most of the changes in the exposome over time and are acknowledged as the Anthropocene era. At the apex of this epoch, particularly in the last century, dramatic and unprecedented rates of change in human activity were followed by the rapid consumption of resources and reduction in biodiversity [189], coinciding with the global increase in noncommunicable diseases and GI cancers. Many components of Earth’s system have changed radically, and the consequences of recent increases in human population and enterprises have also imposed changes on the landscape, climate, and biosphere [190].

It is also important to recognize that the role of social self-regulation patterns in an individual’s development constitutes a generation-specific process and, therefore, is ultimately society-specific [191]. Analogously, from a biological point of view, data on host-associated microbiomes, for example, indicate that host phenotypes, that is, individual phenotypes, can be transmitted between hosts along with the microbiome [192] and that the social connectivity of the individual is capable of modifying both the composition and traits of the microbiome [193], both of which are consistent with the metacommunity theory. As proposed recently, the application of ecological theory to host-microbiome communities represents an opportunity to incorporate transmission and scale-related matters, such as developmental, behavioral, and evolutionary feedback between the individual and microbiome, into a single conceptual framework, which is critically important for interpreting microbiome variation [194,195]. This could represent a means for integrating macro-ecosystems with an individual, regarded as an ecosystem. 

### 5.3. Lifestyle, Noncommunicable Diseases, and Syndemic 

To effectively understand the role of individual behaviors and social determinants in cancer development, fundamental points need to be addressed. From a socioeconomic standpoint, globalization is defined as a process of convergence and unification of the socioeconomic systems of different countries. Based on this thought, globalization could be considered not only as the development of trade activities between countries, but also as a qualitatively new stage in the process of internationalization in which the development and unification of the institutional structure of different states also influence objects of culture and everyday life, defined as lifestyle [196]. Therefore, a Westernized lifestyle, representing a hegemonic societal structure, should be interpreted not only by focusing on individual behaviors, but also by considering an integrative approach to structural determinants of health disadvantage and risk [197]. Nevertheless, structural interventions are obviously more challenging than individual behavioral approaches. However, although dietary patterns and physical activity have usually been regarded as individual lifestyle choices, in 2008, the Commission on Social Determinants of Health of the World Health Organization elaborated a report comprising daily living and working conditions in a broader context of structural determinants to promote health equity [198]. 

Next, it is also essential to revise the meaning of the so-called noncommunicable diseases as potentially inadequate. Previously, another group of investigators suggested binding these diseases based on their common upstream drivers. They proposed the expression, “socially transmitted conditions,” stressing the anthropogenic and socially contagious nature of diseases. Therefore, socially transmitted conditions are driven by urbanization, industrialization, poverty, and the availability of tobacco, alcohol, processed foods, etc., in addition to physical inactivity. Although the suggested new label should not exempt individuals from responsibility for their own lifestyle choices, it might underscore the available limited choices determined by the social environment [75].

Finally, regarding the spread of progressively more common lifestyles in the context of overlapping chronic noncommunicable disorders or socially transmitted conditions, in which GI cancers thrive, it is tempting to think of the globalization of these disorders in a syndemic framework. Analytical inference estimating disease risks, geographical and temporal distributions, and biological, social, and structural factors can contribute to clarifying the potential syndemic nature of the investigated diseases [199]. The recent observation of the co-occurrence of COVID-19 with pre-existing epidemics, including cancer, diabetes, and HIV, for instance, has shown that patients with compromised immune systems have had a clear increase in morbidity and mortality [200]. Considering the global rise of GI cancers as part of a syndemic context, we may guide future efforts to evaluate and incorporate these multiple levels of impact into clinical practice to improve health care, with a special focus on prevention. 

## 6. Study Limitations and Future Perspectives

### 6.1. Limitations of the Current Study

Conducting a review involving the major GI cancers is a challenging task, with implicit limitations. Although several common aspects have been raised in this study, important specific differences among GI cancers do exist, and some have been highlighted here. Nevertheless, comparing different studies, with distinct methodologies, may render data difficult to interpret, especially when considering different cancers. Attempts to identify common molecular, genetic, and epigenetic alterations in GI cancers are not straightforward due to cellular and tissue specificities and differences in microenvironmental and macroenvironmental features. For example, the infectious nature underlying certain types of GI cancers, such as *H. pylori* infection, and viral hepatitis, apparently stem from exclusive epidemiological and pathophysiological backgrounds. In addition, genetic syndromes predisposing to CRC or PC, for instance, also run independent courses compared to the sporadic counterparts of these cancers. Such examples should call attention to the peculiarities of GI cancers, which may not allow for a common rational-specific clinical screening and management of those affected by these disorders and their at-risk relatives, cohabitants, or contacts. Therefore, running a systematic review to respond to a particular question was not appropriate in this study as we were mainly interested in identifying common aspects shared by major GI cancers and mapping knowledge gaps. In fact, this review did not aim to generate a critically appraised response to a specific question, but rather to open a broad discussion on the increasing global incidence of GI cancers and its potential association with factors regarded as noncommunicable, which might guide novel and integrative research projects for directing highly effective public health policies. 

### 6.2. Practical Implications and Research Needs

Although the methodological approach used in this study does not allow objective guidance on GI cancers from a clinical and policy-making standpoint, the issues raised in the current study would direct future systematic reviews and clinical investigations, with innovative approaches. An improved understanding of the multidirectional interactions between macro-ecosystems and the individual, also regarded as an ecosystem, including the potential dysfunctions of the intrinsic gut microbiota induced by different lifestyle aspects, may greatly help prevent diseases. For such complex integrative tasks, it is possible that a multiomics-like approach, already showing a relevant role in cancer diagnosis, survival analysis, and response to treatment [201,202], may also be applied for investigating potentially new and unexplored associations underlying GI cancer pathogenesis. Multiomics approach has recently provided novel insights into the biological mechanisms behind gene–environment interactions [203,204]. In this line, a recent study with multiomics profiling identified several associations revealing potential biological responses and sources of exposure in early life, including signatures for diet, chemical compounds, trace elements, and weather conditions, among others [205]. In addition, recent technological breakthroughs, such as artificial intelligence and machine learning, developed over the past few years [206], may become important tools for supporting the analysis of datasets not restricted to genomics, epigenomics, proteomics, transcriptomics, metatranscriptomics, and other molecular profiling data, but also incorporating social determinants, epidemiological, and lifestyle data, among others. 

At this point, we propose a reappraisal of crucial concepts, such as lifestyle, including the Westernized lifestyle, and a revised definition of noncommunicable health conditions. Analogous to applying the ecological theory to host-microbiome communities, contributing to merging distinct fields into a single conceptual framework to understand microbiome variation, we need to discuss the communicability of societal processes and their impact on the individual. Recognizing the syndemic context in which multiple factors synergize to foster GI cancer development might also impact future epidemiological and pathogenesis studies, including an improved understanding of the role of stress and burnout. Using a novel integrative approach might contribute not only to preventing GI cancers by redefining current health policies, but also to building a sustainable societal structure with relatively fewer health disparities.

## 7. Conclusions

Multiple geosocial factors have been associated with the global rise in chronic noncommunicable diseases, including GI cancers, in the last century. Common societal transformations, usually involving socioeconomic development, have been followed by sociocultural changes in the population progressively agglomerated in urban centers, resulting in changes in the microbiome and genetic and epigenetic modifications. As the Westernized lifestyle spreads globally, changes in the microbiota, constituting an industrial or Westernized gut microbiota, are accompanied by a proinflammatory profile that underlies the rise of most noncommunicable chronic diseases, including cancers. Therefore, it is conceivable to think of an increase in GI cancers as part of a dynamically changing exposome; however, it is progressively very globalized and less diverse.

## Data Availability

Not applicable.
